# A combinatorial approach to the restriction of a mouse genome

**DOI:** 10.1186/1756-0500-6-284

**Published:** 2013-07-22

**Authors:** Leonid V Bystrykh

**Affiliations:** 1 Laboratory of Ageing Biology and Stem Cells, European Research Institute for the Biology of Ageing, University Medical Center Groningen, University of Groningen, Antonius Deusinglaan 1, 9700 AD, Groningen, The Netherlands

**Keywords:** DNA fragmentation, Genome restriction, Randomness of genome

## Abstract

**Background:**

A fragmentation of genomic DNA by restriction digestion is a popular step in many applications. Usually attention is paid to the expected average size of the DNA fragments. Another important parameter, randomness of restriction, is regularly implied but rarely verified. This parameter is crucial to the expectation, that either all fragments made by restriction will be suitable for the method of choice, or only a fraction of those will be effectively used by the method. If only a fraction of the fragments are used, we often should know whether the used fragments are representative of the whole genome. With a modern knowledge of mouse, human and many other genomes, frequencies and distributions of restriction sites and sizes of corresponding DNA fragments can be analyzed *in silico*. In this manuscript, the mouse genome was systematically scanned for frequencies of complementary 4-base long palindromes.

**Findings and conclusions:**

The study revealed substantial heterogeneity in distribution of those sites genome-wide. Only few palindromes showed close to random pattern of distribution. Overall, the distribution of frequencies for most palindromes is much wider than expected by random occurrence. In practical terms, accessibility of genome upon restriction can be improved by a selective combination of restrictases using a few combinatorial rules. It is recommended to mix at least 3 restrictases, their recognition sequences (palindrome) should be the least similar to each other. Principles of the optimization and optimal combinations of restrictases are provided.

## Background

Fragmentation of genomic DNA is very usual step in many protocols aimed at genomic analysis. For instance, studies of DNA methylation employ a genomic restriction protocol with a pair of enzymes, one of which is sensitive to the methyl cytosine in the context of a CG di-nucleotide [1-3]. Telomere length can be measured by use of a combination of for instance two restriction enzymes [4] with the expectation that the longest fragment in the digest will be the telomeric region. A reduced representation approach employs the idea that a randomly selected fraction of DNA fragments adequately represent the whole genome, yet the size of the genomic sample can be substantially reduced [5-8]. A strategy of restriction site associated markers (RAD) for high throughput genotyping is another example of this concept [9]. A few methods aim for maximal accessibility of the genome fragments for detection of some particular genomic elements, for instance retro elements [10-13] or retroviral vectors integrating into the genome [14-16].

In all those kinds of applications it is important to know the cost of deviation from the expected pattern of genomic fragmentation. Issues of genomic bias from randomness can be largely ignored in case of, for instance, restriction-based analysis of DNA methylation: they are exclusively aimed at CpG analysis rather than a randomly sampled genome, the restriction schemes used work well with CpG rich loci but miss large parts of CpG poor regions. In the case of reduced representation, biased genome sampling might affect the calculated genome diversity and phylogeny as a consequence [17]. In the case of genome accessibility, the requirements of the genomic restriction are the highest due to a risk of missing the genomic element in question and drawing incorrect conclusions as a consequence. In the search for endogenous retro elements, we deal with hundreds of those elements across the entire genome, therefore missing a fraction of those might be acceptable. In the case of an analysis of retroviral integration sites, a relatively small number of integrations per cell are generally expected. Therefore the risk and the cost of missing those few might be very high. Yet a common practice of using particular restriction schemes is highly empirical. For a long time, only one restriction enzyme was used for the recovery of retroviral integration sites [18]. It wasn’t until 2007 that Harkey et al., [14] confronted this practice and initiated a search for the optimal combination of restriction enzymes, since a single enzyme restriction was claimed to be ineffective. Ideally all fragments should be as close as possible to the preferred average. An average fragment size should fit the requirements of the assay. Note that studies performed on the analysis of the endogenous retro elements use combinations of 6-base enzyme restrictases [12,13], which are generally very rare cutters falling below 1 cut per 1 kb. Those authors claim a rather broad window of detection, namely up to 4 kb long fragments, therefore the restriction they used would serve the purpose. In a retroviral integration site analysis, the distance of the integrated vector to the restriction site is recommended to be in a range of 20–200 bp [15]. In practice this means that restriction with an enzyme which has an average frequency in the range 5–50 cuts per 1 kb will serve the purpose. Frequent 4-base cutters formally suit those requirements on average. However, it is equally important to ensure that restriction is positionally close to random, as defined for instance in [19]. Until now, this point was rarely expressed or taken in consideration. Since DNA is highly non-random and variable in local frequencies of particular nucleotides is highly unlikely to expect random distribution of restriction sites for any particular restrictase across genome. In the field of retroviral integration site analysis, authors have attempted to circumvent the genome accessibility problem by combining 4-base recognizing restriction enzymes either empirically [16,20], or using a combination of the most frequent cutters [15]. Those combinations are claimed to reach >95% of the whole mouse genome. Although increasing the average restriction frequency might be helpful, this alone is not sufficient to ensure improvement along the entire genomic sequence. It is important to verify that genomic loci void of some particular restriction site will be cut by another enzyme added to the combination and eventually reduce the variation of restricted fragment sizes to the minimum. Alternatively, if the added enzyme would cut at the same loci as another enzyme from the selected enzyme mixture, such a combination will probably be rather counter-effective. It is important therefore to check whether the spectrum of restriction fragments used fits the requirements of the analysis. This inevitably leads to the question how close the distribution of any enzyme or a combination of enzymes is to the random distribution (since an option of making equal fragments is not realistic). If it is too wide, how can it be reduced in practical terms? To illustrate this, all possible complementary palindromes were scanned along the mouse genome, those sites were analyzed for frequencies and randomness of distribution both for each site separately and in combinations. The analysis revealed that distribution of all 4-base long restriction sites is wider than expected by random. A few simple rules can be followed to achieve combinations of restriction sites which fit to a random model. None of the combinations could create a narrower distribution of restriction sites than expected by random.

## Findings

### Fitness to the Poisson distribution

**Figure 1:**
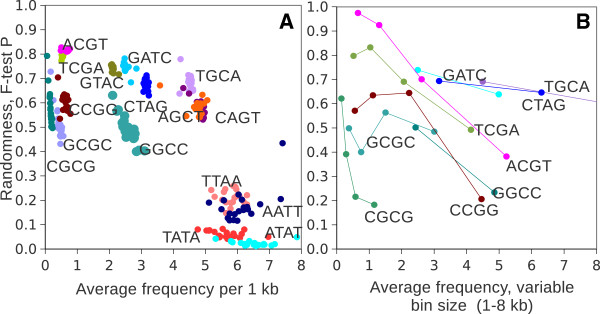
**Frequencies of occurrence and randomness of 4-base palindromes across mouse genome.** 2D plot of randomness, measured as an F-test P value versus average frequencies of tetra-palindromes on all mouse chromosomes **(A)** and trends of randomness for non-AT rich palindromes upon changing bin size and average frequencies per bin therefore **(B)**. Note that GATC, CTAG, TGCA remain quite stable, whereas CG-containing palindromes are moving towards the area of AT-rich palindromes.

**Figure 2:**
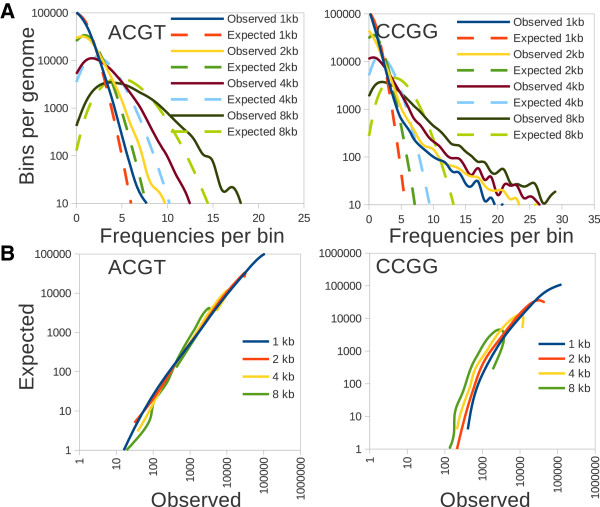
**Example of deviation from randomness for ACGT and CCGG.** Broad shoulders detected upon changing bin size and average frequency per bin **(A)**, non-linearity in expected versus observed frequencies plot **(B)**. Note that linearity of such a plot became worse upon increased bin size and corresponding frequency of restriction.

At present detailed description of mouse and other genomes can be found at several WEB sites, such as NCBI (http://www.ncbi.nlm.nih.gov/genome), Ensembl (http://www.ensembl.org/index.html), or UCSC (http://genome.ucsc.edu). Since the mouse genome is almost completely assembled, frequencies of all possible restriction sites can be mapped with a high precision. This work is mainly focused on an analysis of 4-base long palindromic sequences. Naturally 2^4^ = 16 combinations of complementary palindromes are possible since the last two bases always depend on the combination of the 1st two bases. All chromosomes were scanned for each palindrome, and frequencies of all palindromes per 1 kb non-overlapping windows along the entire chromosome were recorded and further analyzed. Each restriction site showed a rather variable average, varying from 8.25 sites/kb for ATAT on chromosome X to 0.06 sites/kb for CGCG on chromosome Y. For each palindrome, estimated frequencies of cuts were compared with the random hypothesis (Poisson distribution). From the results shown on a Figure 1A is clear that AT-rich palindromes are the most frequent cutters and they deviate the most from the random distribution. Balanced palindromes (those which contain all 4 bases) tend to fit the best to the random test across all frequencies of restrictions, CG-rich palindromes show reasonable randomness but low frequencies of occurrence. The way experimentally found frequencies deviate from the Poisson model is very uniform: Poisson systematically underestimates both low and high frequencies of the distribution (Figure 2A), in other words: the real distribution is systematically wider than expected by random. This deviation can be better visualized in a kind of a “lasso” plot: by plotting observed and predicted frequencies of cuts against each other (Figure 2B). Ideally if observed and predicted frequencies are equal, such a plot should generate a straight line. Instead, it shows a “lasso”-like curve. Importantly, in the case of low cutters, the shoulders of the distribution are smaller, which can cause a better fit to the Poisson when low efficiency cutters are compared to high efficiency enzymes (Figure 1A). To test this, the bin size was varied from 1 kb to 2, 5, 10, 25 kb respectively and the fitness of the lower frequency palindromes to the random model was repeatedly measured (Figure 1B). By increasing bin size from 1 kb to 8 kb (and consequently increasing the average frequency per bin), P values from an F-test became slightly worse for balanced, non-CG containing palindromes (GATC, CTAG, TGCA). Among unbalanced palindromes, a profound change in F-test P value was recorded. In fact the difference between AT and GC rich sites appeared to be due to the differences in frequencies only. When the bin size was increased, CG rich palindromes showed very comparable frequencies per bin and randomness values as found for AT-rich palindromes. To conclude: CG-rich and AT-rich palindromes, although different in frequencies, are not different in their randomness, rather the F-test is sensitive to the frequencies instead.

### Similarity metric by di-nucleotide count

**Figure 3:**
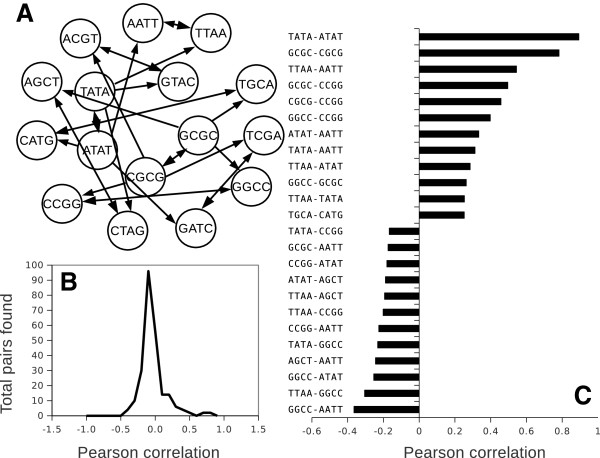
**Relationship between similarity of palindromes and correlation in their restriction frequencies. A**, Similarity network between all complementary palindromes. All edges connect palindromes similar by 2 to 3 di-nucleotide combinations. **B**, Pearson correlation frequencies for all pairs of palindromes on chr1. **C**, Top- and bottom-12 correlations from all pairs on chr1. Note, that the highest positive correlations are found between similar palindromes, whereas negatively correlating palindromes are of an opposite type.

In addition to classification of palindromes as AT-rich, GC-rich and balanced, it is useful to introduce more a precise measure of similarity or difference between palindromes. Since all of them are composed of complementary pairs of bases, a comparison of di-nucleotide composition in each palindrome is a naturally suitable metric. Each palindrome can contain up to 3 different di-nucleotides, for instance the AGCT palindrome contains AG, GC, and CT di-nucleotides. As is shown in Figure 3A, many palindromes have variable similarity to others by having 2 to 3 di-nucleotides in common. For each of 16 possible palindromes, there is at least one highly similar palindrome with 2 di-nucleotides in common.

The same metric was applied for a list of currently known 6-base restriction sites (Table 1). Important difference between 4-base cutters and 6-base cutters is that whereas tetra-palindromes fall naturally in 3 different categories, AT-rich, CG- rich and balanced, hexa-palindromes cannot be fully balanced. Instead, all variety of 6-base sites (totally 55 known restriction sites) falls into 4 groups: AT-rich (e.g. TTTAAA), CG-rich (like CGGCCG), but also AT-enriched (CTTAAG) and GC enriched (CTCGAG).

**Table 1:** Pearson-based combinations of known 6-mer restriction enzymes

**Palindrome**	**Name example**	**Average restriction frequency/10 kb**	**1st best choice**	**Pearson correlation**	**Similarity count**	**2nd best choice**	**Pearson correlation**	**Similarity count**
AACGTT	AclI	0.516	CAATTG	-0.067	2	GGATCC	-0.063	0
AAGCTT	HindIII	3.374	TCCGGA	-0.056	0	GCCGGC	-0.054	1
AATATT	SspI	6.991	CAGCTG	-0.264	0	CTGCAG	-0.257	0
ACATGT	PciI	3.793	AGATCT	-0.137	1	GGATCC	-0.120	1
ACCGGT	AgeI	0.216	AATATT	-0.096	0	TTATAA	-0.079	0
ACGCGT	MluI	0.089	AGTACT	-0.067	2	GATATC	-0.059	0
ACTAGT	SpeI	1.485	AGATCT	-0.080	2	ATCGAT	-0.075	0
AGATCT	BglII	3.614	TTTAAA	-0.188	0	GCATGC	-0.145	1
AGCGCT	AfeI	0.352	AATATT	-0.169	0	CATATG	-0.135	0
AGGCCT	StuI	2.345	AATATT	-0.244	0	TTATAA	-0.232	0
AGTACT	ScaI	2.686	CAGCTG	-0.183	2	CTGCAG	-0.141	2
ATCGAT	ClaI	0.471	TTTAAA	-0.178	0	CAGCTG	-0.141	0
ATGCAT	AvaIII	3.520	AGATCT	-0.136	1	GGTACC	-0.129	0
ATTAAT	AseI	4.695	CTGCAG	-0.202	0	CAGCTG	-0.189	0
CAATTG	MfeI	2.166	CTGCAG	-0.154	2	GCATGC	-0.126	3
CACGTG	AcvI	0.847	AATATT	-0.176	0	CATATG	-0.138	2
CAGCTG	PvuII	3.435	AATATT	-0.264	0	TTATAA	-0.191	0
CATATG	NdeI	3.594	AGGCCT	-0.151	0	CTGCAG	-0.138	2
CCATGG	NcoI	3.056	ATTAAT	-0.152	1	AATATT	-0.145	1
CCCGGG	XmaI	0.566	ATTAAT	-0.183	0	TTATAA	-0.165	0
CCGCGG	KspI	0.111	AATATT	-0.115	0	TTATAA	-0.107	0
CCTAGG	AvrII	2.034	TTTAAA	-0.208	1	TTATAA	-0.160	1
CGATCG	PvuI	0.036	TCATGA	-0.060	3	TTATAA	-0.059	1
CGGCCG	EagI	0.127	CATATG	-0.107	0	AATATT	-0.105	0
CGTACG	PspLI	0.049	AATATT	-0.082	1	TTTAAA	-0.074	1
CTCGAG	XhoI	0.469	CATATG	-0.118	0	TTATAA	-0.116	0
CTGCAG	PstI	3.221	AATATT	-0.257	0	TTATAA	-0.205	0
CTTAAG	AflII	2.347	ATCGAT	-0.094	0	GGATCC	-0.090	0
GAATTC	EcoRI	3.073	CAGCTG	-0.132	0	GCATGC	-0.111	1
GACGTC	ZraI	0.237	AATATT	-0.101	0	TCATGA	-0.085	2
GAGCTC	SacI	2.308	AATATT	-0.171	0	ATTAAT	-0.145	0
GATATC	EcoRV	1.850	CAGCTG	-0.162	0	CTGCAG	-0.154	0
GCATGC	SphI	2.001	AGATCT	-0.145	1	GATATC	-0.142	1
GCCGGC	NaeI	0.289	AATATT	-0.181	0	ATTAAT	-0.148	0
GCGCGC	BsePI	0.177	TTATAA	-0.111	0	AATATT	-0.108	0
GCTAGC	NheI	1.157	AATATT	-0.139	1	ATTAAT	-0.109	1
GGATCC	BamHI	1.989	TTTAAA	-0.201	0	ATTAAT	-0.140	1
GGCGCC	KasI	0.251	ATTAAT	-0.150	0	AATATT	-0.149	0
GGGCCC	ApaI	1.153	AATATT	-0.188	0	ATTAAT	-0.186	0
GGTACC	KpnI	1.184	TTTAAA	-0.188	1	ATGCAT	-0.129	0
GTATAC	SnaI	1.511	AGATCT	-0.076	1	GGTACC	-0.067	3
GTCGAC	SalI	0.128	AGTACT	-0.043	1	TCATGA	-0.040	3
GTGCAC	VneI	1.674	AATATT	-0.179	0	ATTAAT	-0.144	0
GTTAAC	HpaI	1.253	ATCGAT	-0.098	0	AGATCT	-0.075	0
TACGTA	SnaBI	0.333	CCTAGG	-0.057	1	ATCGAT	-0.051	1
TCATGA	BspHI	3.576	GGCGCC	-0.112	0	GCTAGC	-0.102	0
TCCGGA	AccIII	0.363	AATATT	-0.146	0	TTATAA	-0.142	0
TCGCGA	NruI	0.043	ATGCAT	-0.060	1	CATATG	-0.053	0
TCTAGA	XbaI	3.145	GGCGCC	-0.069	0	CCGCGG	-0.059	0
TGATCA	BclI	2.721	CTGCAG	-0.098	2	AGCGCT	-0.090	0
TGCGCA	AviII	0.377	TTATAA	-0.109	0	AATATT	-0.102	0
TGGCCA	MscI	2.658	AATATT	-0.200	0	ATTAAT	-0.165	0
TGTACA	BsrGI	3.332	GGTACC	-0.077	3	TCCGGA	-0.070	0
TTATAA	PsiI	5.286	AGGCCT	-0.232	0	CTGCAG	-0.205	0
TTCGAA	AsuII	0.407	TTATAA	-0.055	2	GTATAC	-0.033	0
TTTAAA	DraI	10.198	CCTAGG	-0.208	1	GGATCC	-0.201	0

Note: Full set of correlations is available in the Additional file 1: Table S3.

### Correlations between palindrome frequencies along the chromosome

**Figure 4:**
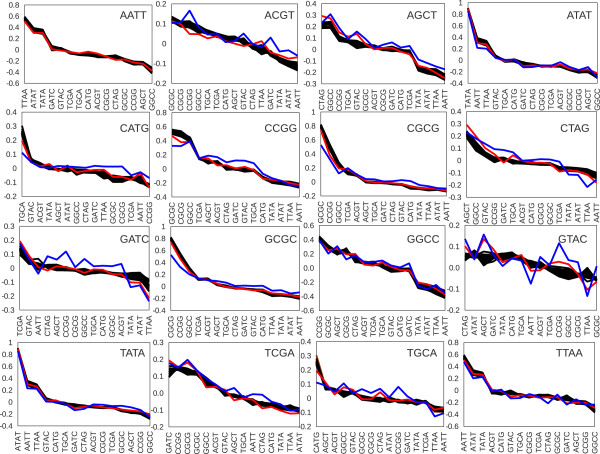
**Correlation between all 4-base palindromes on all mouse chromosomes.** The most deviating from the common trend were ChrX (red line) and ChrY (blue line).

Cross correlation of all tetramer palindromes across mouse chromosome 1 revealed that an average of those correlations slightly shifted into negative region (Figure 3B), the positive shoulder is longer than the negative one. Note that a correlation is analyzed through tens of thousands data points and even small correlation values are highly significant. For instance, a correlation of 0.05 along one of the smallest chromosome 19 collects approximately 60000 data points and a corresponding p-value < 0.0001. A selection of the lowest 10 and the highest 10 correlations (Figure 3C), which constitutes 19% of all correlated pairs, appeared to be enriched with unbalanced palindromes (note that the number of balanced and unbalanced palindromes is equal). Of those, palindromes similar in their di-nucleotide composition and type of enrichment (GC or AT) show significant positive correlations. The complementary palindromes from opposite enrichment groups tend to show negative correlation. Analysis of correlations was repeated for all chromosomes. Similar results were obtained for the same palindrome along the whole mouse genome (Figure 4, Additional file 1: Table S1). Note that only X and Y chromosomes deviated most drastically from the common trend whereas all autosomes showed highly similar results. In practical terms, this finding ensures that analysis of one autosome is already well representative of the whole mouse genome. It saves computational time as well as simplifying visualization of the data. The trends found by di-nucleotide context and palindrome enrichment type could be further followed and analyzed in more detail during the selection of a series of best anti-correlating palindromes. Obviously, the purpose of such selection is to test the possibility of finding combinations of various restriction sites where a desired average frequency is achieved together with minimal bias and maximally possible randomness of distribution.

### A negative correlation strategy

**Figure 5:**
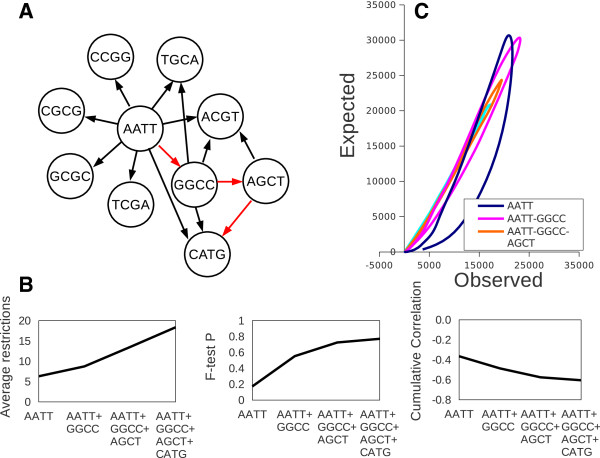
**Example of a combination by negative correlation strategy. A**, Choices of negatively correlating palindromes (with a threshold < −0.05) along consequently made choices, AATT, GGCC, AGCT, CATG. **B**, Parameters for the selected group gradually reaching their maximum/minimum values. **C**, a “lasso” plot shows improved linearity upon gradual addition of each subsequent palindrome to the mixture.

We can try to begin with each of the 14 palindromes for which restriction enzymes are available and follow the most negatively correlating site as the best candidate for a mixture. At this stage ATAT and TATA palindromes were excluded from analysis since they do not represent any known restriction enzyme. An algorithm will stepwise select for the most negatively correlating palindrome and consecutive count of combined frequencies of already selected palindromes. An example of such a strategy is shown in Figure 5 for the selected sequences AATT, GGCC, AGCT, and CATG. A network graph shows that the total number of negatively correlating palindromes gradually decreases upon addition of the next palindrome to the mixture. Whereas AATT is negatively correlating with 8 different palindromes, addition of the third palindrome, AGCT shows only two candidates, ACGT and CATG. Upon addition of the last palindrome, CATG, no more candidates to the mixture were revealed (in a threshold < −0.05). Note that the correlation value between the selected group and every subsequently added palindrome is decreasing (and the cumulative correlation value is decreasing as well, Figure 5B). At the same time, randomness (fitness to the Poisson distribution) of the combined palindrome frequencies is asymptotically increasing, as shown in the “lasso” plot (Figure 5C), namely a plot of observed frequencies versus expected gradually became linear. A full screen of all possible starting palindromes revealed that, in fact, good randomness is already achieved in a mixture of 3 palindromes. Further additions are useful mainly to satisfy average restriction frequency.

**Figure 6:**
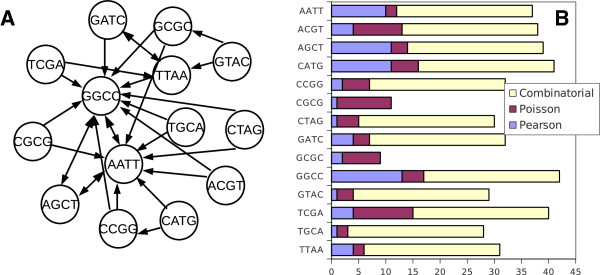
**Typical choices and total numbers of unique sets in different strategies of combining restriction enzymes. A**, a network representation of the first two choices made by the most negative correlation strategy when using all possible palindromes. Note that AATT and GGCC are the most frequent choices. **B**, a histogram count of all used palindromes using different strategies. Note that Pearson and Poisson strategies used 14 trials in total, the combinatorial approach used 60 trials (total unique combinations).

When starting with different palindromes, out of 14 trials (with all possible palindromes as the 1st choice) only 10 unique combinations were found (for details see Additional file 1: Table S2). In this series there is a strong effect of the most frequent and most biased palindromes, like TTAA, AATT, which always require CG rich palindromes for compensation, the most frequent compensating palindrome was found to be GGCC. A network of 1st 2 choices made by the negative correlation strategy illustrates this trend (Figure 6A) and summarizes a “popularity contest” among different palindromes. Figure 6B shows the total frequency of palindromes in a complete series of 5 out of 14. Similarly, AATT, AGCT, CATG and GGCC appeared to be the most frequent choices for a Pearson-based strategy.

During this analysis, it was found that there is an approximate solution for the laborious recalculation of correlation coefficients upon addition of a new palindrome to the mixture (it was actually done for all discussed examples). In fact the next best palindrome can be found using the primary paired correlation table by estimating the weighted (by restriction frequency) average between two previously added components (group or single palindrome, more details in Additional file 1: Table S1, page example), the palindrome with the most negative correlation to the group can be selected as the next best candidate to the mixture.

### Use of an F-test for finding palindrome combinations

Considering such a small number of found combinations listed above we could try some alternative strategies. For instance, a search by Pearson correlations works well with biased distributions, but it is probably less sensitive when dealing with more balanced and randomized palindromes. With this idea in mind, the F-test was tried instead. Screening through all starting palindromes showed good agreement with AT rich and balanced palindromes. One new combination with a good randomness was revealed (Additional file 1: Table S2). However, this algorithm appeared to be completely nonfunctional in the case of low frequency GC rich palindromes, such as CGCG, TCGA, ACGT and GCGC. Apparently such enrichment occurs due to the problem of the F-test estimation (mentioned earlier in this text): with increased frequency of the restriction, the F-test value became worse, which hinders the search for the optimal randomization. This approach is inferior to the selection by negative correlation and can be recommended for balanced and frequent palindromes only.

### Comparison with combinatorial model

**Figure 7:**
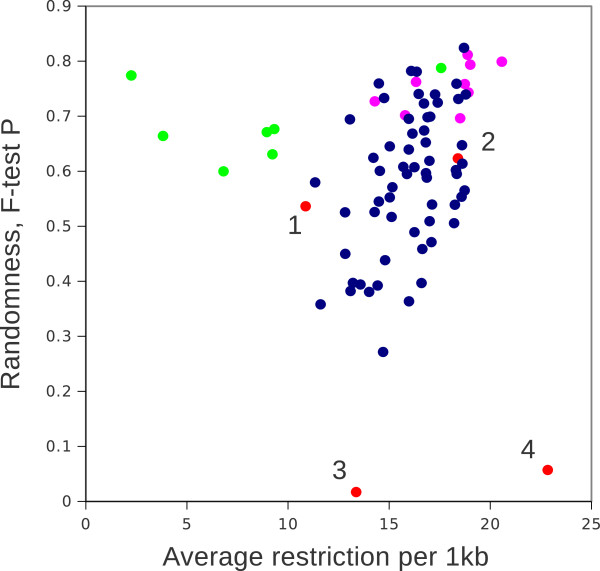
**Combinations of palindromes generated by different approaches.** Pink dots, selection by negative Pearson correlation; green, selection by F-test; blue, all combinations by 5 from 14 palindromes with 14 di-nucleotides used, red, combinations from literature:1, Harkey et al. [14], 2, Bystrykh et al. [21], 3, Biasco et al. [20], 4, Gabriel et al. [15], which is identical to simplified Wang et al. [16]. Details in a Additional file 1: Table S1.

It was already mentioned above (Figure 3C) that the resulting combinations based on a Pearson correlation revealed interesting combinatorial rules: similar palindromes will be naturally excluded and the most dissimilar palindromes will be selected (as they are negatively correlating). We can follow this trend in more detail by counting cumulative frequencies of all possible di-nucleotides within previously found combinations of palindromes (Additional file 1: Table S2). Note that since each palindrome can contain 3 different di-nucleotides at maximum the non-redundant set of restriction sites (no repeated use of di-nucleotides is allowed) should not be higher than 5. Although a maximum of 15 di-nucleotide combinations out of 16 possible could be reached, the results show that maximal coverage of possible di-nucleotides in a set of 15 palindromes is 14. Out of 8568 combinations screened, a list of 60 combinations containing 14 different di-nucleotides was generated. A variation of total restriction frequencies varied from 1 to 19 per 1 kb bin size. Selective testing for randomness with the F-test demonstrated that these are quite credible combinations quite closely resembling the sets generated by the negative correlation strategy (Figure 7). Overall, the cloud of all combinations found is broad in terms of an average frequency and fitness to the random distribution. Out of 60 total combinations, all except CGCG and GCGC were equally represented in these series (Figure 6B). A selection by Pearson correlation is localized with few more examples found by combinatorial approach. Both of them are substantially better than combinations found using F-test fitness to Poisson distribution. Only one alternative combination with high total frequency of restriction was found in a set of F-test generated series, the same combination was the best regarding randomization of di-nucleotides.

### Previously suggested combinations

As already mentioned before, a use of a single cut for studies of retro elements is problematic, since neither frequency nor accessibility of fragments are at their optimum. In the case of retroviral integration site analysis, one restriction enzyme, cutting at TTAA was used for quite some time. It is only since 2007 [14], when a mixture of enzymes was suggested in order to maximize genome accessibility. Figure 7 and Additional file 1: Table S2 demonstrate average frequencies and randomness of a few published combinations. A mix by Wang et al. [16] is unnecessarily redundant. After removing redundancy it became identical to a mix suggested by Gabriel et al. [15], who were aiming on the maximal restriction frequency ignoring a di-nucleotide bias in selecting two AT-rich enzymes, one balanced (also AT containing) and no GC rich enzymes. As a consequence, such a mixture is highly non-random and the spread of the cut fragment size will be unnecessarily broad: it will systematically undercut GC-rich regions, many of which are situated in promoter regions, which are potential targets for integration of retroviral vectors. Similarly, an empirical set by Biasco et al. [20] is almost the same in average frequency as given by Harkey et al., (2007). However, the difference in randomness of those mixtures is dramatic. Extensive restriction of AT rich regions by mixing the most frequent restrictases faces a risk of generating too small fragments (primarily in AT rich regions) to be used for site identification. On the contrary, the addition of GC-rich restrictases, albeit with a minimal increment in average frequency, will substantially improve randomness of the cut and therefore will improve the overall chances of detecting integrated vectors. Note that a mixture suggested in our recent review [21] was purely based on the combinatorial principle before the current analysis was done.

### Use of 6-base restrictases

Whereas 4-base restrictases show frequencies of restrictions in a range from 0.05 to 8 cuts per 1 kb, 6- base cutters are approximately 10 times less frequent (see data for chr1 in Additional file 1: Table S3 for details). Therefore their use must be justified after critical evaluation of the method of choice. General trends among 6-base cutters are similar to those found and described above for tetra-palindromes. AT-rich restriction sites are the most frequent, balanced 6-cutters do not exist. Instead AT- or GC- enriched palindromes show intermediate frequencies, and GC-rich sites are the least frequent along the genome. If two restrictases would be combined, the combinatorial rule still stands: AT-rich palindromes a best matching to GC-rich (as can be revealed by negative correlations). All other palindromes, however, behave less predictable (regarding combinatorial rule) and more elaborate concepts should be developed. For practical purpose, if any selected combinations will be implemented, names of known 4- and 6-base restriction endonucleases and corresponding palindromes are provided in a Additional file 1: Table S4.

## Discussion

Fragmentation of genomic DNA by restriction remains a routine element in modern genomic studies. Unfortunately, the selection of particular restriction enzymes for the application is often empirical and not subjected to any optimization or validation. An issue of prediction and distribution of nucleotide dimers, restriction sites and small words was previously discussed in the literature. Originally prediction of restriction sites was based on a single-base probabilistic rule [22]. In the case of microbial genomes, near to random distributions were suggested for restriction sites [19] or small words [23]. However, a bias in di-nucleotide frequencies [24] and genomic k-mers [25] were also documented in large genomes. Therefore the assumption of randomness should be made carefully for analysis of short DNA sequences, including restriction sites. The concern that restriction with a particular enzyme or mixture of enzymes might substantially deviate from random did not spread far outside the field of theoretical genome biology. For instance, in studies of genomic retro elements, restriction of DNA is performed with empirical combinations of 6-base restrictases [12,13], and no issue of randomness is addressed. Note that 6-base cutters show considerably lower frequencies compared to 4-cutters (see some examples in [21]) and therefore inferior in frequencies to the examples of combinations given in this paper. In fact use of 6-base cutters for fragmentation of large genomes should be theoretically justified, otherwise not recommended. In the field of population genetics, the problem of biased sampling and its possible drawbacks is already introduced [17]. In a recent publication it was anticipated that the performance of genome sequencing itself depends on representative and random fragmentation [26]. An issue of non-optimal fragment size is one of many problems recognized in chromosome conformation capture protocols [27,28]. Yet, in many of those publications authors have used 1 or 2 empirically selected 6- or 4-base cutters to create genomic fragments. In similar studies [6,7] only a single 4-base restriction enzyme was used for genome digestion. Van Tassel et al., [6] selected their 4-base cutter by computer analysis to minimize the appearance of repeated elements in their virtual fragments. This selection makes sense to improve efficiency of mapping. However, as was already mentioned here, a single cut strategy is always inferior to the combination of restrictases regarding its randomness. Some genome restriction based protocols have already reached clinical studies, for instance gene therapy treated patients. In those studies, the problem of accessibility has been already identified and discussed [14,15]. However authors still focus their attention on an average restriction frequency without much consideration of the randomness. As a result, authors almost empirically suggested mixtures of enzymes with low to medium randomness (examples are shown in Figure 7). Note that those few suggested combinations are further replicated in more recent clinical studies. To conclude, the problem of a proper fragmentation of genomic DNA and its verification for randomness remains an unexplored option for further improvement. As it is demonstrated here, none of the restriction sites are fully random. However, some restrictases are more random than others. Combinations of restrictases presented in this paper provide the best random distribution across the mouse genome.

The F-test was used here for the assessment of randomness, which although superior to the Chi-squared test [29], should be used with care. It is sensitive to the average frequency of the distribution. Nevertheless the F-test served the purpose quite well, namely it helped to compare different restrictases, and also helped to establish how combinations of restriction enzymes could improve the randomness of genomic restriction.

Although the combinatorial principles revealed might look puzzling and unexpected, the explanation is quite simple. The reason similar palindromes correlate is because they correlate to the regions enriched in subsequences which they contain. If they contain similar subsequences, for instance similar di-nucleotides, they will tend to be positively correlating. For the same reason, complementary di-nucleotides (AT vs GC, AA vs GG etc.) will tend to show negative correlation. This combinatorial rule is essentially based on Markov chains models, it is well reproducible in a different genomic context (see consistency of correlations across autosomes), it is also resistant to SNP variations and other minor mutations in a population of considered species. In the case of balanced palindromes, this rule will be more problematic, because 4 bases cannot be enriched all at the same time: any enrichment also means loss of complementary bases or di-nucleotides. Consequently it will hamper enrichment of corresponding subsequences. Although the combinatorial principle was found to be useful as a guideline, some deviations from it were also evident. For instance, CG-containing palindromes tend to correlate with each other more than would be expected from similarity measurements. Mutually exclusive pairwise palindromes with relatively low frequency might co-occur in a bigger set of palindromes due to the dominating effect of more frequent and biased palindrome.

There can be many factors indirectly causing non-randomness of restriction sites along the genome. It might reflect an evolutionary expansion of DNA by self-duplication, selective pressure in coding regions and in CpG islands for CG- rich palindromes. Some evolutionary pressure can be expected for ATAT and TATA sequences due to their similarities to TATA boxes. It is, however, difficult to expect direct selective pressure on the distribution of restriction sites themselves (unless a clear functional demonstration of those sequences will be provided). Instead, an effect of local enrichment for sub-words, for instance di-nucleotides, presented in GC or AT rich palindromes is most likely. This selectivity of genomic landscape fundamentally breaks the principle of equal chance along the genome and this might be a primary cause of deviation from the Poisson model. Analytically, the Poisson model (see also Methods) can be adjusted to such situations, if *λ* is the average frequency of an event and *k* is the expected exact frequency, we can consider *λ* as being a function of *k* (equation can be found in the Methods). The notion that the genomic landscape is primarily responsible for non-randomness of restriction sites is an important example how genomic non-randomness in general can be interpreted beside other existing explanations. As an example, Falconer et al. [30] found a wider distribution of segregating DNA template strands than predicted by a random distribution (very similar to the case of non-randomness found in this paper). Such non-randomness was interpreted as selective process, disregarding the alternative option of having non-random (selective) genomic background for the fairly random process of template segregation. Those two options, not one, should be considered for further experimental verifications.

During preparation of our recent review [21] on a subject of clonal analysis of hematopoietic cells, we briefly suggested the combinatorial principle of mixing restrictases for optimal genome fragmentation, which was hypothetical at that time. This paper provides the necessary background to this concept in sufficient detail and demonstrates how this principle helps in analysis and finding the best combinations of restrictases. Although mainly tetra-nucleotide palindromes were studied here, the strategy described can be applied to restrictases of any length.

## Conclusions

Enzymatic restriction is a very frequent step in fragmentation of genomic DNA. A question of the randomness of restriction is often expected or implied, but rarely verified. A genome-wide restriction analysis of 4-base cutters revealed significant deviation from randomness for practically all tested restriction enzymes. The randomness can be improved by combining separate restrictases in the mixture. A combinatorial approach is probably the simplest but most effective principle to achieve such improvement.

## Methods

For the mouse genome, the Bioconductor (version 2.9) metadata package Bsgenome.Mmusculus.UCSC.mm10 for R version 2.15 was used which consists of the mmu10 assembly from UCSC (based on C57BL/6 J mouse strain). This package was used in conjunction with R (version 2.14.1) to scan the genome for the presence of all 16 possible complement palindromes in non-overlapping bins of 1 kb in size. All bins with N < 500 were included in the resulting table. For each complement palindrome, fitness to the Poisson distribution per chromosome was calculated using a custom Python script and Gnumeric spreadsheet (in Linux). For a random model, a Poisson distribution was used as following:

$$f\left(k;\lambda \right)=\lambda^{k}e^{-\lambda }/k!$$

Where *λ* is observed average, *k* is exact number of events expected at given average, *f*(*k*;*λ*) is a probability of such event.

Randomness of restriction was tested by comparing for observed and Poisson predicted data sets using the F-test as commonly described [27]. A p-value for the two-tailed hypothesis test comparing the variances of two populations, in our case palindrome frequencies and Poisson distribution, was calculated using the built in F-test function in the Gnumeric spreadsheet program. When used in the text, this is referred as an F-test P value or F-test P on the plot. Pearson correlation was calculated along the entire chromosome of choice using a custom script in python, using a double-pass strategy and the equation;

$$r=\sum \left(X_{i}-\bar{X}\right)\left(Y_{i}-\bar{Y}\right)/\sqrt{\sum {\left(X_{i}-\bar{X}\right)}^{2}}\sqrt{\sum {\left(Y_{i}-\bar{Y}\right)}^{2}}$$

The probability of correlation was assessed using the t-test transformation.

$$t=r\sqrt{\left(n-2\right)}/1-r^{2}$$

and an online probability calculator http://www.statstodo.com/TTest_Pgm.php.

Through the text all palindromes are mentioned by their DNA sequence. Names of the corresponding restriction endonucleases are provided in the Additional file 1: Table S4.

## Competing interests

Author declares no competing interests.

## Supplementary Material

Additional file 1: Table S1 Pearson correlation data for all tetra palindromes all mouse chromosomes. **Table S2**: Selection of all palindromic combinations using 3 different approaches. **Table S3**: Pearson correlation data for all tetra palindromes mouse chromosome 1. **Table S4**: Commercial names for all 4- and 6- base restriction endonucleases. Additional files. The Supplementary files S1, S2, S3, S4 are deposited in an open access repository https://easy.dans.knaw.nl/ui/datasets/id/easy-dataset:53177. http://www.persistent-identifier.nl?identifier=urn:nbn:nl:ui:13-d9zq-rm. Click here for file
